# Diagnosis of Alzheimer’s disease in Brazil: Supplementary exams

**DOI:** 10.1590/S1980-57642011DN05030004

**Published:** 2011

**Authors:** Paulo Caramelli, Antonio Lúcio Teixeira, Carlos Alberto Buchpiguel, Hae Won Lee, José Antônio Livramento, Liana Lisboa Fernandez, Renato Anghinah, Group Recommendations in Alzheimer’s Disease and Vascular Dementia of the Brazilian Academy of Neurology

**Affiliations:** 1 Department of Internal Medicine, School of Medicine, Federal University of Minas Gerais, Belo Horizonte MG, Brazil; 2 Department of Radiology, School of Medicine, University of São Paulo, São Paulo SP, Brazil; 3 Institute of Radiology, Hospital das Clínicas, School of Medicine, University of São Paulo and Hospital Sírio-Libanês, São Paulo, SP, Brazil; 4 Medical Investigation Laboratory (LIM 15), School of Medicine, University of São Paulo, São Paulo SP, Brazil; 5 Department of Basic Health Sciences, Federal University of Health Sciences Foundation of Porto Alegre, Porto Alegre RS, Brazil; 6 Referral Center for Cognitive Disorders (CEREDIC), Hospital das Clínicas, School of Medicine, University of São Paulo, São Paulo SP, Brazil

**Keywords:** consensus, guidelines, diagnosis, supplementary exams, Alzheimer’s disease, Brazil

## Abstract

This article presents a review of the recommendations on supplementary exams employed for the clinical diagnosis of Alzheimer’s disease (AD) in Brazil published in 2005. A systematic assessment of the consensus reached in other countries, and of articles on AD diagnosis in Brazil available on the PUBMED and LILACS medical databases, was carried out. Recommended laboratory exams included complete blood count, serum creatinine, thyroid stimulating hormone (TSH), albumin, hepatic enzymes, Vitamin B12, folic acid, calcium, serological reactions for syphilis and serology for HIV in patients aged younger than 60 years with atypical clinical signs or suggestive symptoms. Structural neuroimaging, computed tomography or – preferably – magnetic resonance exams, are indicated for diagnostic investigation of dementia syndrome to rule out secondary etiologies. Functional neuroimaging exams (SPECT and PET), when available, increase diagnostic reliability and assist in the differential diagnosis of other types of dementia. The cerebrospinal fluid exam is indicated in cases of pre-senile onset dementia with atypical clinical presentation or course, for communicant hydrocephaly, and suspected inflammatory, infectious or prion disease of the central nervous system. Routine electroencephalograms aid the differential diagnosis of dementia syndrome with other conditions which impair cognitive functioning. Genotyping of apolipoprotein E or other susceptibility polymorphisms is not recommended for diagnostic purposes or for assessing the risk of developing the disease. Biomarkers related to the molecular alterations in AD are largely limited to use exclusively in research protocols, but when available can contribute to improving the accuracy of diagnosis of the disease.

## Abstract (pt)

Este artigo apresenta revisão das recomendações sobre os exames complementares empregados para o diagnóstico clínico de doença de Alzheimer (DA) no Brasil, publicadas em 2005. Foram avaliados de modo sistemático consensos elaborados em outros países e artigos sobre o diagnóstico de DA no Brasil disponíveis no PUBMED ou LILACS. Os exames laboratoriais recomendados são hemograma completo, creatinina sérica, hormônio tíreo-estimulante, albumina, enzimas hepáticas, vitamina B12, ácido fólico, cálcio, reações sorológicas para sífilis e, em pacientes com idade inferior a 60 anos, com apresentações clínicas atípicas ou com sintomas sugestivos, sorologia para HIV. Exame de neuroimagem estrutural, tomografia computadorizada ou – preferencialmente – ressonância magnética, é indicado na investigação diagnóstica de síndrome demencial, para exclusão de causas secundárias. Exames de neuroimagem funcional (SPECT e PET), quando disponíveis, aumentam a confiabilidade diagnóstica e auxiliam no diagnóstico diferencial de outras formas de demência. O exame do líquido cefalorraquidiano é preconizado em casos de demência de início pré-senil, com apresentação ou curso clínico atípicos, hidrocefalia comunicante e quando há suspeita de doença inflamatória, infecciosa ou priônica do sistema nervoso central. O eletroencefalograma de rotina auxilia no diagnóstico diferencial de síndrome demencial com outras condições que interferem no funcionamento cognitivo. A genotipagem da apolipoproteína E ou de outros polimorfismos de susceptibilidade não é recomendada com finalidade diagnóstica ou para avaliação de risco de desenvolvimento da doença. Os biomarcadores relacionados às alterações moleculares da DA ainda são de uso quase exclusivo em protocolos de pesquisa, mas quando disponíveis podem contribuir para maior precisão diagnóstica da doença.

## Introduction

In recent decades, major advances have been made in research toward diagnosing Alzheimer’s disease (AD), particularly studies on early detection. Consensus and recommendations of societies of medical specialties in the field are generally updated periodically to stay abreast of the impact of new diagnostic methods and instruments for potential introduction into routine clinical practice.

The aim of the present module was to review and update recommendations governing supplementary exams for diagnosing AD in Brazil. The modalities of the exam were grouped into six categories. The critical review of the scientific literature and proposed preliminary recommendations were the responsibility of each of the authors contributing to this module, all of whom are experts in their specific area of knowledge. The recommendations were subsequently debated by the members of the groups to reach the final recommendations. The final recommendations were then presented, discussed and voted on at meetings with all other colleagues involved.

## Blood tests

Laboratory blood tests have traditionally been used in the context of the propedeutic on dementia syndrome to exclude possible secondary causes. The American Academy of Neurology (AAN) recommends only the investigation of Vitamin B^12^ deficiency and of hypothyroidism in the initial propedeutic on patients with clinically suspected dementia.^1^ Considering the specificities of the Brazilian population, the preliminary guidelines of the Science Department of Cognitive Neurology and Aging of the Brazilian Academy of Neurology (ABN) for diagnosing Alzheimer’s Disease (AD) proposed a significantly more comprehensive list of exams for the assessment of patients with dementia syndrome. This included full blood count, sera levels of urea, creatinine, free thyroxin (T4), Thyroid Stimulating Hormone (TSH), albumin, hepatic enzymes (TGO, TGP, Gama-GT), Vitamin B^12^, calcium, serological reactions for syphilis, and HIV serology in patients younger than 60 years of age.^2^ Closely reflecting these recommendations, the 2010 European Federation of Neurological Societies (EFNS) guidelines also suggest a more extensive list of exams which includes folic acid concentrations.^3^

Over the past two decades however, blood (serum, plasma and blood cells) has also been considered a potential source of biomarkers for diagnosing AD.^4^ Ease of obtention of patient blood compared with cerebrospinal fluid (CSF) render plasma and serum biomarkers particularly attractive for use in research and routine clinical practice.

The majority of these studies have focused on investigating single or small series of molecules related to the physiopathologic processes of AD such as amyloid genesis, inflammation and oxidative stress.

The level of tau protein in the blood is extremely low and thus falls below the detection threshold of most tests employed. Studies have explored the potential of the amyloid-β 1-42 and amyloid-β 1-40 peptides as biological marker candidates of AD with conflicting results, showing their ability or otherwise to discriminate AD patients from healthy controls.^5,6^ Some evidence suggests that a steady fall in plasma amyloid-β peptides is associated with progressive cognitive decline in the course of AD but further studies are needed to correlate these findings.^7^ Thus, unlike the assessment of tau protein or amyloid-β peptides in CSF, serum or plasma, levels of these molecules appear to have little clinical value.

Regarding the molecules related to inflammatory processes (C-reactive protein, interleukin 6 or IL-6, soluble receptor of tumor necrosis factor alpha or TNF-alpha) oxidative stress (isoprostane), neurotrophic factors (brain derived neurotrophic factor or BDNF) among others, the value of these as reliable biomarkers of AD is not yet clear. This is owing to conflicting evidence and results from studies involving only a small number of patients. In addition, it is noteworthy that these molecules are not exclusively linked to the physiopathology of AD since they can be altered in other diseases such as in the case of inflammatory molecules during infectious conditions.

More recently, strategies not aimed at target molecules such as the simultaneous analysis of multiple molecules and proteomic analysis have been employed with promising results. Two studies in this area are worthy of mention. Ray et al.^8^ showed that the combination of 18 plasma signalling proteins including cytokines, chemokines and trophic factors were able or differentiate AD subjects from controls with around 90% accuracy whereas in the study by O’Bryant et al.,^9^ a combination of 23 serum proteins, predominantly involved with inflammation, but not the same as those employed in the study by Ray et al.,^8^ were shown to provide 91% sensitivity and 80% specificity for diagnosing AD.

**Recommendations** - (1) Laboratory blood tests (complete blood count, serum creatinine levels, TSH, albumin, hepatic enzymes, Vitamin B12, folic acid, calcium, serological reactions for syphilis, and serology for HIV in patients aged younger than 60 years with atypical clinical signs or suggestive symptoms) should be conducted to check for secondary causes of dementia syndrome (Standard). Based on clinical discretion, other laboratory exams can also be ordered. (2) Based on the current state of research knowledge, no plasma or serological biomarkers can be recommended for use in diagnosing AD or monitoring its progression (Rule). (3) Tests measuring circulating blood levels of tau protein or amyloid-β peptides are also not indicated for use (Rule).

## Structural neuroimaging

Computed tomography (CT) and magnetic resonance (MR) of the brain are used in the initial assessment of patients with dementia. CT can be used to rule out secondary causes and reversible dementia such as subdural hematomas, tumors or normal pressure hydrocephaly. However, MR, given its superior ability to reveal anatomic detail and detect alterations is the first method of choice, except when its use is contra-indicated. In addition, MR plays a central diagnostic role for some dementia types such as vascular dementia^10^ and Creutzfeldt-Jacob disease,^11,12^ besides contributing to the identification of frontotemporal lobe degeneration.^13^

Reduced volume of the hippocampus, entorhinal cortex and posterior cingulate are early signs of AD.^14-19^ Some studies have also shown that atrophy of this region can identify patents with MCI that convert toAD.^17,20^ At a later stage, these volume reductions extend to affect the frontal, parietal and temporal neocortices.^21,22^

The most straightforward approach for assessing hippocampal atrophy is through visual inspection of the hippocampus by an experienced examiner on coronal plane images. This technique offers from 80 to 85% sensitivity and specificity in differentiating individuals with AD from cognitively normal subjects and a slightly lower sensitivity for diagnosing MCI.^24,25^ MRI scans also prove superior for assessing temporal medial atrophy. However, should this method be unavailable or contra-indicated, the use of coronal orientation (or coronal reconstruction) on CT scans using coronal plane images is recommended whenever possible,^26^ because it can better assess temporal medial atrophy.

Volumetric assessment can be manual or automated, and offers slightly enhanced sensitivity (90%) and specificity (91%) for differentiating AD and MCI cases from controls^27^ while temporal medial atrophy is a valid diagnostic criteria for diagnosing AD in research studies (group comparison). It should be emphasized however, that volumetric data must be normalized for application in clinical practice, particularly for individual assessment of less impaired cases.^28^ Nevertheless, longitudinal assessment, preferably performed at the same institution (due to variability of acquisition and processing techniques where manual volumetric assessments have the added pitfall of inter-examiner variability), can be potentially valuable as a diagnostic aid. The rates of global brain and hippocampal atrophy are sensitive markers of the progression of neurodegeneration and are increasingly used in clinical trials with therapies which can potentially modify disease evolution.^28^

Proton magnetic resonance spectroscopy (MRS) is an MR application enabling non-invasive *in vivo* assessment of metabolites.^29,30^ It is considered a functional neuroimaging method but is discussed under this section together with the other parameters and data obtained for MR. The most frequent findings of MRS studies in AD are reductions in N-acetyl-L-aspartate (Naa) and its ratios (Naa/creatine (Cr) and Naa/water) and increases in myoinositol (mI) and its ratios (mI/Cr and mI/water), with increased mI and ratios being an earlier finding. The mI/Naa ratio, which combines two of the most significant metabolic alterations in AD, is considered important in detecting the disease.^31-34^

However, the metabolic alterations outlined above are non-specific.^35-36^ Thus, correlation with clinical data and preferential analysis of early or typical sites of early impairment may serve to increase the accuracy of the method. The posterior cingulate is one such region commonly targeted in many studies and is technically easier to evaluate and reproduce than the hippocampus.^37-39^

MRS has less validation as a marker of AD than temporal medial atrophy, even in research studies,^28^ and although its findings allow accurate discrimination between AD patients and controls, and contribute to disease staging,^40^ broad normatization of normal values is needed for individual application in routine clinical practice. However, when hallmark features are detected in individuals with cognitive decline, MRS findings can serve to corroborate the clinical diagnosis.

Other MR volumetric techniques such as diffusion weighted MRI (DWI), tractography by diffusion tensor MRI (DTI), magnetization transference, brain perfusion MRI, arterial spin labeling (ASL) and functional MRI are markers with a lesser degree of validation in research protocols and have no established role in clinical practice.^41,42^

**Recommendations** - (1) Structural neuroimaging exams, CT or preferably MR, are indicated for diagnostic investigation of dementia syndrome to rule out secondary causes (Standard). (2) The identification of temporal mesial atrophy on MRI scans, by visual analysis and manual or automated volumetry, contribute to the diagnosis of AD in clinical practice (Practice Option), although is of greater value for use in group comparisons within research protocols. (3) MRS can be recommended for research protocols.

## Molecular and functional neuroimaging

### Markers

Currently, the diagnosis of neurodegenerative conditions can be based on two main classes of biomarkers:

(1) “pathologic signature” biomarkers; and
(2) biomarkers of neuronal degeneration and synaptic dysfunction.

Pathologic signature markers constitute markers of amyloid-β plaque deposits in neural tissue on positron emission tomography (PET). The presence of β-amyloid deposits are known to precede the emergence of clinically-confirmed AD by years or even decades.^43^ Results of antemortem studies in patients with MCI and even populations diagnosed with AD, correlated with those of necropsy studies, have confirmed a strong association of the *in vivo* presence of this biomarker with the clinical disease or evolution/conversion to AD.^44,45^

The markers of progressive neuronal degeneration are based essentially on determination of synaptic dysfunctions and functional disconnections by detection of regional perfusion and metabolism deficits in bilateral posterior temporo-parietal cortex and precuneus/posterior cingulate gyrus, respectively, by single-photon emission computed tomography (SPECT) and PET. Studies on correlation with necropsy findings are available, showing high diagnostic accuracy of these biomarkers when correlated with the characteristic anatomopathological substrates involved in AD.^46^

### Clinical applications of biomarkers: limitations and indications

#### “PATHOLOGICAL SIGNATURE” BIOMARKERS

Despite evidence of a correlation between the *in vivo* presence of the amyloid-β protein on PET and the diagnosis of AD in the dementia, MCI or even pre-clinical states,^47^ the role of these biomarkers as a tool for early detection of the disease in routine clinical practice remains unclear.^48^ Several factors limit the routine use of these biomarkers, namely:

- Availability and cost in Brazil.
- Absence of standardized qualitative and quantitative criteria for accurately differentiating among high, low and intermediate probability diagnoses.
- Definition of its value as a prognostic indicator of future conversion to dementia. Additionally, the typical time interval which elapses between detection and development of dementia is not yet known.
- Many studies use matched group analyses in which transposition to individual-based analyses is not yet well defined.
- Absence of a proven therapeutic arsenal enabling reversal of, or control of evolution to, the dementia stage of AD, especially in pre-clinical or MCI phases.

#### BIOMARKERS OF NEURONAL DEGENERATION

There is currently a body of evidence, including correlation with necropsy findings, that demonstrates high accuracy in diagnosing AD by means of determination of metabolic and perfusional deficits in bilateral association cortex, including the precuneus and posterior cingulate.^49^ Patients with MCI presenting with these functional deficits on functional neuroimaging as an indirect indicator of neuronal degeneration and principally, synaptic dysfunction, shall be categorized as converters in contrast to patient groups which have no deficit in regional blood flow on SPECT (rCBF) or regional glucose consumption deficit on PET.^50^ The progressive cognitive decline seen in AD is strongly associated with the presence of synaptic dysfunction which in turn is directly correlated with PET/SPECT findings.^51^ However, these findings are not strictly specific and may be observed in association with other neurological conditions (such as Parkinson’s disease and vascular dementia). Therefore, indication of these techniques should invariably be made as a supplement to clinical diagnosis, which remains the gold standard for AD diagnosis.

Another controversial aspect is the choice between PET and SPECT, given that the former offers 15 to 20% greater accuracy but is significantly more costly and with limited availability in Brazil. Therefore, supplementary indication remains conditioned on clinical judgement, taking into account the availability of the technique and the socio-economic situation.

**Recommendations** - 1) When available, “pathological signature” biomarkers can be employed in investigation protocols or in clinical therapy trials. In clinical practice, their use can contribute to greater accuracy diagnosing AD in both dementia and MCI phases (Rule). (2) Biomarkers of neuronal degeneration (SPECT and PET) when available, increase diagnostic reliability in clinically well-defined cases of AD and also assist in the differential diagnosis of other types of dementia (Rule).

## Cerebrospinal fluid exam (CSF)

The CSF exam comprises the supplementary propedeutic on the diagnosis of various causes of dementia. It is extremely useful for identifying infectious dementia conditions affecting the central nervous system such as neurosyphilis, neurocysticercosis, neuro-Aids (dementia-Aids complex), herpetic meningoencephalitis, chronic meningitis, Creutzfeldt-Jakob disease; in dementia conditions of neoplastic, paraneoplastic and lymphoproliferative diseases; in dementia conditions of inflammatory and auto-immune diseases; as well as in hydrocephalus especially normal pressure hydrocephalus with application of the “tap-test”.^2,52-55^

In AD, there are biomarkers appearing in CSF that determine a “pathological signature” of the disease. This entails the measures of two alterations:

(1) reduction in amyloid-β 1-42 protein, the main component of neuritic plaques;
(2) increase in tau and phosphorylated tau proteins, due to neuronal degeneration associated to intracellular accumulation of neurofibrillary tangles.^48,56-58^

Reduction in amyloid-β 1-42 protein and increase in tau and phosphorylated tau have a sensitivity and specificity ranging from 85% to 90% for diagnosing AD.^56^

Temporally, pre-clinical and pre-dementia phases of AD can be observed by reduced amyloid-β 1-42 protein levels in CSF.^59^ In a later phase, albeit still clinically asymptomatic, the neuronal degeneration markers tau and phosphorylated tau protein can also be detected. Similarly, these markers are also changed in patients with MCI evolving to AD.^60^

Interpretation of these biomarkers in CSF should be done carefully and set against the clinical condition of the patient. This is important since the classic profile of alterations across all CSF biomarkers is often lacking. Future multicentric studies are needed before implementation of the exam in routine clinical practice.^61,62^

**Recommendations** - (1) The CSF is indicated in the investigation of pre-senile onset dementia (before 65 years of age) in cases with atypical clinical presentation or course, communicant hydrocephaly and the presence of any evidence or suspicion of inflammatory, infectious or prion disease of the central nervous system (Standard). (2) Levels of amyloid-β 1-42 peptide and tau and phosphorylated tau proteins in CSF can be employed in research protocols or clinical therapy trials. In clinical practice, its use can contribute to greater accuracy diagnosing AD in both dementia and MCI phases (Rule).

## Electroencephalogram (EEG) and evoked potentials

Visual analysis of routine EEG is a useful method to aid differential diagnosis of dementia types,^63,64^ distinguishing between dementia syndrome, cognitive complaints and psychiatric disorders. EEG can also aid diagnosis of Creutzfeldt-Jakob disease, suggest the possibility of toxic-metabolic disorder or transient epileptic amnesia.^3^ The most common findings in AD are slowed background frequency with increased delta and theta bands, and reduction or abolition of the alpha frequency band.^65^ However, these changes are generally only visible on EEG in moderate and advanced stages of AD. There is an inverse correlation between degree of cognitive impairment and strength of electrical activity at high frequencies (alpha and beta) on EEG.^66^ A reduction in the alpha band and increase in theta, plus lower mid-range frequencies, are characteristic electroencephalographic findings of patients with AD, but EEGs can be normal at early stages of the disease in up to 14% of cases.^67^ The accuracy of electroencephalographic diagnosis of AD patients versus healthy controls with similar demographics reported by different studies varies widely.^67^ EEG revealing patterns of diffuse abnormalities alone are more frequently associated to AD whereas those showing diffuse and focal alterations suggest AD and/or other forms of dementia.^68^

Since the very first quantitative EEG studies,^69,70^ both spectral and statistical analyses have been applied to the method. Lower alpha and beta activity has been observed in a number of studies conducted over the last few decades.^71-73^ In addition, the alpha rhythm could serve as a potential diagnostic marker,^73^ since there is a reduction in alpha frequency to 6.0-8.0 Hz in patients with mild AD. Another highly sensitive aspect in EEG is the base spectral analysis which is associated with the clinical diagnosis of AD. The sensitivity of spectral analysis has been found to range from 71% to 81% in various studies^72,74,75^ and correlates significantly with neuropsychological tests.^75^

Another feature offered by EEG is coherence analysis (Coh) which assesses the level of covariance among spectral measurements obtained by a pair of electrodes. High COh has been taken as evidence of structural and functional connections among cortical regions.^76^

In guidelines produced by the Brazilian Medical Association (AMB) and the Brazilian Society of Clinical Neurophysiology (SBNC),^77^ EEG is recognized as an established method for assessing dementias. In addition, frequency analysis represents a valuable tool for improving the detection of slow waves. This analysis can show increased theta wave activity and reduced alpha and beta waves in AD patients compared with healthy individuals.^78^ Frequency analysis is also a predictor of the development of cognitive impairment, independently of clinical parameters.^79^ Moreover, there is a strong correlation between EEG activity and cognitive brain functions quantified by specific assessment scales.^79^ The use of a combination of these EEG parameters with cognitive assessment instruments is recommended to improve dementia detection.

With regard to evoked potentials, delayed P300 latency is considered the best parameter for electrophysiological diagnosis of cognitive decline and dementia. However, the wide inter-individual variation (approximately 50 milliseconds) limits its diagnostic reliability in initial phases of AD, since this changes can also occur in depression, schizophrenia and other dementia types.^2,63^

**Recommendations** - (1) The use of routine EEG is an established supplementary method for differential diagnosis of dementia syndrome from other conditions impairing cognitive functioning such as epilepsy, toxic-metabolic and infectious encephalopathies (Standard). EEG is an important tool for diagnosing Creutzfeldt-Jakob disease (Standard). (2) EEG is not helpful for early diagnosis of AD (Standard). (3) Event-related evoked potentials (example P300, N400) are recommended for use in the research setting only.

## Genetic study

On the genetic front, rare dominant autosomal mutations indicating early onset of AD (before 65 years of age) with complete penetrance is associated to three genes: amyloid precursor protein (APP),^80^ presenilin 1 (PSEN1),^81^ and presenilin 2 (PSEN2) genes.^82^ Mutations of the APP gene located at chromosome 21 are found within or adjacent to areas which codify the amyloid-β peptide and account for less than 5% of familial AD cases.^83^ Mutations in PSEN1 and PSEN2 genes located at chromosome 14 and 1, respectively, codify the proteins of highly conserved membrane needed for activity of the γ-secretase enzyme which cleaves the APP protein. Mutations in PSEN1 are responsible for the majority of cases of familial AD whereas mutations in PSEN2 are less frequent.^83,84^

The ε4 allele of apolipoprotein E (APOE), a susceptibility variant with common and incomplete penetrance, significantly increases the risk of developing late-onset AD (after 65 years of age).^85-87^ The APOE gene, located at chromosome 19, has three common allelic forms: ε2 (occurs in 8% of the white population), ε4 (in 15%) and ε3 (in 75%). The presence of the ε4 allele triples the risk of developing the disease and individuals homozygous for ε4 have a 12-fold greater chance of developing AS than ε3 individuals. The presence of the ε2 allele however, is a protective factor against AD.^87^ Similar allelic and genotypic distribution, besides association of the presence of the ε4 allele with AD diagnosis, were also found in population-based and case-control studies in Brazil.^88-92^ APOE is involved in cholesterol transport and formation of the amyloid-β by as yet unknown mechanisms.^87^ Approximately 42% of individuals with AD do not carry the ε4 allele of the APOE gene.^93^

Numerous publications compiled by the AlzGene database report associations between AD and hundreds of supposed risk alleles in other genes.^94^ The neuronal sortilin-related receptor (SORL1) has been genetically associated to late-onset AD in a population of heterogeneous ethnicity in the United States.^95,96^ A recent meta-analysis showed evidence of association of genetic susceptibility polymorphisms located at chromosome 1 (CR1), chromosome 7 (PICALM) and 8 (CLU), although without the same impact odds ratio of the APOE.^97^ Cholesterol is believed to modulate central processes in the pathogenesis of AD. The association of the APOE, CH25H, CLU, LDLR, and SORL1genes with AD could be mediated by cholesterol-related mechanisms or by direct effects of these proteins on amyloid-β metabolism.^98^

In general, all people with Down’s syndrome (trisomy of chromosome 21) develop neuropathologic markers for AD after 40 years while more than half of this patient group have cognitive decline. This believed to be caused by overexpression of the gene of APP at chromosome 21, leading to increased production of the amyloid-β peptide.^98^

Most individuals with early-onset MCI and mutations in the genes APP, PSEN1 or PSEN2, develop AD, as do individuals with late-onset AD and one or two ε4 alleles of the APOE.^61,99^

### Indication of genetic testing for AD

In general, clinical use of the genetic test for APOE with predictive intent in asymptomatic individuals is not recommended because the presence of the ε4 allele is not necessary nor sufficient to reach a diagnosis of AD.^100,101^ Family history on the other hand, represents a better predictor of risk for AD.^101^ Empirically, first-degree relatives of a single individual with AD have a 20-25% chance of developing the disease during their lifetime compared to 10% for individuals with no family history of the disease.^93^

With regard to the diagnosis of pre-clinical AD, the role of biomarkers for detecting and tracking this stage of the disease is of central importance for the development of effective treatments. In this context, monitoring of carriers of the ε4 allele of the APOE suggests evidence of very early onset synaptic dysfunction (young and middle-aged individuals) on functional neuroimaging studies. It should be underscored that recommendations for diagnosing pre-clinical AD apply exclusively for research purposes, having no clinical implications at present.^62^

The presence of the ε4 allele of the APOE is not sufficiently specific for inclusion in the new criteria for probable AD with a high degree of certainty.^62^ Clinico-pathologic series in which the genotyping of the APOE was estimated were not favorable for introduction of the test for the gene in clinical practice. The sensitivity and specificity of clinical diagnosis alone were 93% and 55%, respectively, whereas for the genotyping of the APOE this was 68% and 65%, respectively.^102^

Although the ε4 allele of the APOE is an important predictive factor for conversion of MCI into AD, its use in clinical practice is not yet established.^99,102^ However, in future studies of potential pre-morbid biomarkers for AD, the inclusion of genetic genotyping is indicated to increase accuracy.^102^

Genetic susceptibility tests for asymptomatic adults at risk for early-onset AD due to APP, PSEN1 and PSEN2 mutations are clinically available. There is a general consensus that these tests should not be performed in childhood.^101^ Moreover, there is consensus that the performing of tests must be preceded by thorough and extensive genetic counseling and assessment of the favorable and unfavorable aspects of disclosure. Monitoring of these individuals who receive genetic information should also be carried out.^3,87,103-105^

**Recommendations** - (1) Genotyping of APOE is not recommended for diagnostic purposes in patients with AD, nor as a predictive factor of developing the disease in individuals that are asymptomatic or who have MCI in clinical practice (Standard). The same holds for other susceptibility polymorphisms described to date (Standard). (2) Investigation of the mutations of APP, PSEN1 and PSEN 2, when available, is recommended in cases of AD with a family history consistent with autosomal-dominant inheritance (Standard).(3) Investigation of mutations of APP, PSEN1 and PSEN 2, when available, in asymptomatic individuals with family member(s) who have genetically-confirmed diagnosis of AD should only be indicated after extensive genetic counseling and with the full consent of the individual (Practice Option).

## GROUP RECOMMENDATIONS IN ALZHEIMER’S DISEASE AND VASCULAR DEMENTIA OF THE BRAZILIAN ACADEMY OF NEUROLOGY

**Amauri B. da Silva** [UNINEURO, Recife (PE)]; **Ana Cláudia Ferraz** [Serviço de Neurologia do Hospital Santa Marcelina (SP)]; **Analuiza Camozzato de Pádua** [Universidade Federal de Ciências da Saúde de Porto Alegre (UFCSPA); Hospital de Clínicas de Porto Alegre (UFRGS) (RS)]; **Ayrton Roberto Massaro** [Instituto de Reabilitação Lucy Montoro (SP)]; **Benito Pereira Damasceno** [Departamento de Neurologia da Universidade Estadual de Campinas (SP)]; **Carla Tocquer** [Universidade Federal do Rio de Janeiro (RJ)]; **Cássio Machado C. Bottino** [Programa Terceira Idade, Instituto de Psiquiatria do Hospital das Clínicas da Faculdade de Medicina da Universidade de São Paulo (FMUSP) (SP)]; **Charles André** [Faculdade de Medicina - UFRJ; SINAPSE Reabilitação e Neurofisiologia (RJ)]; **Cláudia C. Godinho** [Serviço de Neurologia do Hospital de Clínicas de Porto Alegre, Universidade Federal do Rio Grande do Sul (RS)]; **Cláudia Sellitto Porto** [Grupo de Neurologia Cognitiva e do Comportamento da Faculdade de Medicina da USP (SP)]; **Delson José da Silva** [Núcleo de Neurociências do Hospital das Clínicas da Universidade Federal de Goiás (UFG); Instituto Integrado de Neurociências (IINEURO), Goiânia (GO)]; **Denise Madeira Moreira** [Departamento de Radiologia Faculdade de Medicina - UFRJ; Setor de Radiologia - INDC - UFRJ (RJ)]; **Eliasz Engelhardt** [Setor de Neurologia Cognitiva e do Comportamento - INDC - CDA/IPUB - UFRJ (RJ)]; **Elza Dias-Tosta** [Presidente da Academia Brasileira de Neurologia, Hospital de Base do Distrito Federal (DF)]; **Emílio Herrera Junior** [Departamento de Medicina Interna, Faculdade de Medicina de Catanduva (SP)]; **Francisco de Assis Carvalho do Vale** [Universidade Federal de São Carlos (UFSCar), Departamento de Medicina (DMed) (SP)]; **Gabriel R. de Freitas** [Instituto D’or de Pesquisa e Ensino; Universidade Federal Fluminense (RJ)]; **Ivan Hideyo Okamoto** [Departamento de Neurologia e Neurocirurgia; Instituto da Memória - Universidade Federal de São Paulo - UNIFESP (SP)]; **Jerusa Smid** [Grupo de Neurologia Cognitiva e do Comportamento do Hospital das Clínicas da Faculdade de Medicina da Universidade de São Paulo (FMUSP) (SP)]; **João Carlos Barbosa Machado** [Aurus IEPE - Instituto de Ensino e Pesquisa do Envelhecimento de Belo Horizonte; Faculdade de Ciências Médicas de Minas Gerais (FCMMG), Serviço de Medicina Geriátrica do Hospital Mater Dei (MG)]; **José Luiz de Sá Cavalcanti** [Departamento de Neurologia - INDC - UFRJ; Setor de Neurologia Cognitiva e do Comportamento - INDC - UFRJ (RJ)]; **Letícia Lessa Mansur** [Grupo de Neurologia Cognitiva e do Comportamento do Departamento de Neurologia da FMUSP; Departamento de Fisioterapia, Fonoaudiologia e Terapia Ocupacional da Faculdade de Medicina da USP (SP)]; **Márcia Lorena Fagundes Chaves** [Serviço de Neurologia do Hospital de Clínicas de Porto Alegre, Universidade Federal do Rio Grande do Sul (RS)]; **Márcia Radanovic** [Laboratório de Neurociências - LIM27, Departamento e Instituto de Psiquiatria da Faculdade de Medicina da Universidade de São Paulo (FMUSP) (SP)]; **Márcio Luiz Figueredo Balthazar** [Universidade Estadual de Campinas (UNICAMP), Faculdade de Ciências Médicas (FCM), Departamento de Neurologia (SP)]; **Maria Teresa Carthery-Goulart** [Grupo de Neurologia Cognitiva e do Comportamento do Departamento de Neurologia da Faculdade de Medicina da USP; Centro de Matemática, Computação e Cognição, Universidade Federal do ABC (SP)]; **Mônica S. Yassuda** [Grupo de Neurologia Cognitiva e do Comportamento do Departamento de Neurologia da Faculdade de Medicina da USP; Departamento de Gerontologia, Escola de Artes, Ciências e Humanidades da USP (EACH/USP Leste) (SP)]; **Nasser Allam** [Universidade de Brasília (UnB), Laboratório de Neurociências e Comportamento, Brasília (DF)]; **Norberto Anízio Ferreira Frota** [Universidade de Fortaleza (UNIFOR), Serviço de Neurologia do Hospital Geral de Fortaleza (HGF) (CE)]; **Orestes Forlenza** [Laboratório de Neurociências - LIM27, Departamento e Instituto de Psiquiatria da Faculdade de Medicina da Universidade de São Paulo (FMUSP) (SP)]; **Paulo Henrique Ferreira Bertolucci** [Universidade Federal de São Paulo (UNIFESP), Setor de Neurologia do Comportamento - Escola Paulista de Medicina, São Paulo (SP)]; **Regina Miksian Magaldi** [Serviço de Geriatria do Hospital das Clínicas da FMUSP, Centro de Referência em Distúrbios Cognitivos (CEREDIC) da FMUSP (SP)]; **Renata Areza-Fegyveres** [Grupo de Neurologia Cognitiva e do Comportamento do Hospital das Clínicas da Faculdade de Medicina da Universidade de São Paulo (FMUSP) (SP)]; **Ricardo Nitrini** [Grupo de Neurologia Cognitiva e do Comportamento do Hospital das Clínicas da Faculdade de Medicina da Universidade de São Paulo (FMUSP); Centro de Referência em Distúrbios Cognitivos (CEREDIC) da FMUSP (SP)]; **Rodrigo Rizek Schultz** [Setor de Neurologia do Comportamento do Departamento de Neurologia e Neurocirurgia da Universidade Federal de São Paulo, Núcleo de Envelhecimento Cerebral (NUDEC) - Instituto da Memória (UNIFESP) (SP)]; **Rogério Beato** [Grupo de Pesquisa em Neurologia Cognitiva e do Comportamento, Departamento de Medicina Interna, Faculdade de Medicina, UFMG (MG)]; **Sonia Maria Dozzi Brucki** [Grupo de Neurologia Cognitiva e do Comportamento da Faculdade de Medicina da Universidade de São Paulo; Centro de Referência em Distúrbios Cognitivos (CEREDIC) da FMUSP; Hospital Santa Marcelina (SP)]; **Tânia Novaretti** [Faculdade de Filosofia e Ciências, Campus de Marília, da Universidade Estadual Paulista (UNESP) (SP)]; **Valéria Santoro Bahia** [Grupo de Neurologia Cognitiva e do Comportamento do Hospital das Clínicas da Faculdade de Medicina da Universidade de São Paulo (FMUSP) (SP)]; **Ylmar Corrêa Neto** [Universidade Federal de Santa Catarina (UFSC), Departamento de Clínica Médica, Florianópolis (SC)].

## References

[1] Knopman DS, DeKosky ST, Cummings JL (2001). Practice parameter: diagnosis of dementia (an evidence-based
review). Report of the Quality Standards Subcommittee of the American
Academy of Neurology. Neurology.

[2] Nitrini R, Caramelli P, Bottino CM, Damasceno BP, Brucki SM, Anghinah R, Academia Brasileira de Neurologia (2005). Diagnóstico de doença de Alzheimer no Brasil:
critérios diagnósticos e exames complementares. Arq Neuropsiquiatr.

[3] Hort J, O'Brien JT, Gainotti G (2010). EFNS guidelines for the diagnosis and management of Alzheimer's
disease. Eur J Neurol.

[4] Humpel C, Marksteiner J, Ritsner MS (2009). Peripheral biomarkers in dementia and Alzheimer's
disease. The handbook of neuropsychiatric biomarkers, endophenotypes and genes.
Volume III: metabolic and peripheral biomarkers.

[5] Schneider P, Hampel H, Buerger K (2009). Biological marker candidates of Alzheimer's disease in blood,
plasma, and serum. CNS Neurosci Ther.

[6] Song F, Poljak A, Smythe GA, Sachdev P (2009). Plasma biomarkers for mild cognitive impairment and Alzheimer's
disease. Brain Res Rev.

[7] Locascio JJ, Fukumoto H, Yap L (2008). Plasma amyloid beta-protein and C-reactive protein in relation to
the rate of progression of Alzheimer disease. Arch Neurol.

[8] Ray S, Britschgi M, Herbert C (2007). Classification and prediction of clinical Alzheimer's diagnosis
based on plasma signaling proteins. Nat Med.

[9] O'Bryant SE, Xiao G, Barber R (2010). A serum protein-based algorithm for the detection of Alzheimer
disease. Arch Neurol.

[10] Roman GC, Tatemichi TK, Erkinjuntti T (1993). Vascular dementia: diagnostic criteria for research studies:
report of the NINDS-AIREN International Workshop. Neurology.

[11] Tschampa HJ, Kallenberg K, Urbach H (2005). MRI in the diagnosis of sporadic Creutzfeldt-Jakob disease: a
study on inter-observer agreement. Brain.

[12] Collie DA, Sellar RJ, Zeidler M, Colchester AC, Knight R, Will RG (2001). MRI of Creutzfeldt-Jakob disease: imaging features and
recommended MRI protocol. Clin Radiol.

[13] Neary D, Snowden JS, Gustafson L (1998). Frontotemporal lobar degeneration: a consensus on clinical
diagnostic criteria. Neurology.

[14] Convit A, De Leon MJ, Tarshish C (1997). Specific hippocampal volume reductions in individuals at risk for
Alzheimer's disease. Neurobiol Aging.

[15] Jack CR Jr, Petersen RC, Xu YC (1997). Medial temporal atrophy on MRI in normal aging and very mild
Alzheimer's disease. Neurology.

[16] Ball MJ, Fisman M, Hachinski V (1985). A new definition of Alzheimer's disease: a hippocampal
dementia. Lancet.

[17] Fox NC, Warrington EK, Freeborough PA (1996). Presymptomatic hippocampal atrophy in Alzheimer's disease: a
longitudinal MRI study. Brain.

[18] Laakso MP, Soininen H, Partanen K (1998). MRI of the hippocampus in Alzheimer's disease: sensitivity,
specificity, and analysis of the incorrectly classified
subjects. Neurobiol Aging.

[19] Scheltens P, Leys D, Barkhof F (1992). Atrophy of medial temporal lobes on MRI in "probable" Alzheimer's
disease and normal ageing: diagnostic value and neuropsychological
correlates. J Neurol Neurosurg Psychiatry.

[20] Visser PJ, Verhey FR, Hofman PA, Scheltens P, Jolles J (2002). Medial temporal lobe atrophy predicts Alzheimer's disease in
patients with minor cognitive impairment. J Neurol Neurosurg Psychiatry.

[21] McDonald CR, McEvoy LK, Gharapetian L, Alzheimer's Disease Neuroimaging Initiative (2009). Regional rates of neocortical atrophy from normal aging to early
Alzheimer disease. Neurology.

[22] Fox NC, Scahill RI, Crum WR, Rossor MN (1999). Correlation between rates of brain atrophy and cognitive decline
in AD. Neurology.

[23] Korf ES, Wahlund LO, Visser PJ, Scheltens P (2004). Medial temporal lobe atrophy on MRI predicts dementia in patients
with mild cognitive impairment. Neurology.

[24] DeCarli C, Frisoni GB, Clark CM, Alzheimer's Disease Cooperative Study Group (2007). Qualitative estimates of medial temporal atrophy as a predictor
of progression from mild cognitive impairment to dementia. Arch Neurol.

[25] Duara R, Loewenstein DA, Potter E (2008). Medial temporal lobe atrophy on MRI scans and the diagnosis of
Alzheimer disease. Neurology.

[26] O'Brien JT (2007). Role of imaging techniques in the diagnosis of
dementia. Br J Radiol.

[27] Desikan RS, Cabral HJ, Hess CP, Alzheimer's Disease Neuroimaging Initiative (2009). Automated MRI measures identify individuals with mild cognitive
impairment and Alzheimer's disease. Brain.

[28] Frisoni GB, Fox NC, Jack CR Jr, Scheltens P, Thompson PM (2010). The clinical use of structural MRI in Alzheimer
disease. Nat Rev Neurol.

[29] Castillo M, Kwock L, Mukherji SK (1996). Clinical applications of proton MR spectroscopy. AJNR Am J Neuroradiol.

[30] Miller BL (1991). A review of chemical issues in 1H NMR spectroscopy:
N-acetyl-L-aspartate, creatine and choline. NMR Biomed.

[31] Shonk TK, Moats RA, Gifford P (1995). Probable Alzheimer disease: diagnosis with proton MR
spectroscopy. Radiology.

[32] Moats RA, Ernst T, Shonk TK, Ross BD (1994). Abnormal cerebral metabolite concentrations in patients with
probable Alzheimer disease. Magn Reson Med.

[33] Parnetti L, Tarducci R, Presciutti O (1997). Proton magnetic resonance spectroscopy can differentiate
Alzheimer's disease from normal aging. Mech Ageing Dev.

[34] Rose SE, de Zubicaray GI, Wang D (1999). A 1H MRS study of probable Alzheimer's disease and normal aging:
implications for longitudinal monitoring of dementia
progression. Magn Reson Imaging.

[35] Capizzano AA, Schuff N, Amend DL (2000). Subcortical ischemic vascular dementia: assessment with
quantitative MR imaging and 1H MR spectroscopy. AJNR Am J Neuroradiol.

[36] Wardlaw JM, Marshall I, Wild J, Dennis MS, Cannon J, Lewis SC (1998). Studies of acute ischemic stroke with proton magnetic resonance
spectroscopy: relation between time from onset, neurological deficit,
metabolite abnormalities in the infarct, blood flow, and clinical
outcome. Stroke.

[37] Lee HW (2005). Evaluation of Azheimer's disease using magnetic resonance spectroscopy:
comparation between findings in the posterior cingulate and
hippocampi.

[38] Kantarci K, Knopman DS, Dickson DW (2008). Alzheimer disease: postmortem neuropathologic correlates of
antemortem 1H MR spectroscopy metabolite measurements. Radiology.

[39] Schott JM, Frost C, MacManus DG, Ibrahim F, Waldman AD, Fox NC (2010). Short echo time proton magnetic resonance spectroscopy in
Alzheimer's disease: a longitudinal multiple time point
study. Brain.

[40] Engelhardt E, Moreira DM, Laks J, Cavalcanti JL (2005). Alzheimer's disease and proton magnetic resonance spectroscopy of
limbic regions: a suggestion of a clinical-spectroscopic
staging. Arq Neuropsiquiatr.

[41] Stebbins GT, Murphy CM (2009). Diffusion tensor imaging in Alzheimer's disease and mild
cognitive impairment. Behav Neurol.

[42] Smith CD (2010). Neuroimaging through the course of Alzheimer's
disease. J Alzheimers Dis.

[43] Fagan AM, Mintun MA, Mach RH (2006). Inverse relation between AbreItalicoin vivoFechaItalico amyloid
imaging load and cerebrospinal fluid Abeta42 in humans. Ann Neurol.

[44] Jack CR Jr, Lowe VJ, Weigand SD, Alzheimer's Disease Neuroimaging Initiative (2009). Serial PIB and MRI in normal, mild cognitive impairment and
Alzheimer's disease: implications for sequence of pathological events in
Alzheimer's disease. Brain.

[45] Aizenstein HJ, Nebes RD, Saxton JA (2008). Frequent amyloid deposition without significant cognitive
impairment among the elderly. Arch Neurol.

[46] Jagust W (2006). Positron emission tomography and magnetic resonance imaging in
the diagnosis and prediction of dementia. Alzheimers Dement.

[47] Sheline YI, Raichle ME, Snyder AZ (2010). Amyloid plaques disrupt resting state default mode network
connectivity in cognitively normal elderly. Biol Psychiatry.

[48] Hampel H, Frank R, Broich K (2010). Biomarkers for Alzheimer's disease: academic, industry and
regulatory perspectives. Nat Rev Drug Discov.

[49] Silverman DH, Small GW, Chang CY (2001). Positron emission tomography in evaluation of dementia: regional
brain metabolism and long-term outcome. JAMA.

[50] Drzezga A, Lautenschlager N, Siebner H (2003). Cerebral metabolic changes accompanying conversion of mild
cognitive impairment into Alzheimer's disease: a PET follow-up
study. Eur J Nucl Med Mol Imaging.

[51] Terry RD, Masliah E, Salmon DP (1991). Physical basis of cognitive alterations in Alzheimer's disease:
synapse loss is the major correlate of cognitive impairment. Ann Neurol.

[52] Herskovits AZ, Growdon JH (2010). Sharpen that needle. Arch Neurol.

[53] Knopman DS (2011). Tapping into the biology of Alzheimer disease. Neurology.

[54] Machado LR, Livramento JA, Spina-França A, Mutarelli EG (2006). Exame de líquido cefalorraquidiano. Manual de exames complementares em Neurologia.

[55] Marra C (2003). CSF: techniques and complications.

[56] De Meyer G, Shapiro F, Vanderstichele H, Alzheimer's Disease Neuroimaging Initiative (2010). Diagnosis-independent Alzheimer disease biomarker signature in
cognitively normal elderly people. Arch Neurol.

[57] Roe CM, Fagan AM, Williams MM (2011). Improving CSF biomarker accuracy in predicting prevalent and
incident Alzheimer disease. Neurology.

[58] Shaw LM, Vanderstichele H, Knapik-Czajka M, Alzheimer's Disease Neuroimaging Initiative (2009). Cerebrospinal fluid biomarker signature in Alzheimer's disease
neuroimaging initiative subjects. Ann Neurol.

[59] Jack CR Jr, Knopman DS, Jagust WJ (2010). Hypothetical model of dynamic biomarkers of the Alzheimer's
pathological cascade. Lancet Neurol.

[60] Hansson O, Zetterberg H, Buchhave P, Londos E, Blennow K, Minthon L (2006). Association between CSF biomarkers and incipient Alzheimer's
disease in patients with mild cognitive impairment: a follow-up
study. Lancet Neurol.

[61] Albert MS, DeKosky ST, Dickson D (2011). The diagnosis of mild cognitive impairment due to Alzheimer's
disease: recommendations from the National Institute on Aging and
Alzheimer's Association workgroup. Alzheimer's & Dementia.

[62] Sperling RA, Aisen PS, Beckett (2011). Toward defining the preclinical stages of Alzheimer's disease:
recommendations from the National Institute on Aging and the Alzheimer's
Association workgroup. Alzheimer's & Dementia.

[63] Luccas FJC, Anghinah R, Braga NIO (1999). Recomendações para o
registro/interpretação do mapeamento topográfico do
eletrencefalograma e potenciais evocados. Parte II:
correlações clínicas. Arq Neuropsiquiatr.

[64] Sandmann MC, Piana ER, Sousa DS, Bittencourt PRM (1996). Eletrencefalograma digital com mapeamento em demência de
Alzheimer e doença de Parkinson. Arq Neuropsiquiatr.

[65] Lehmann D (1971). Multichannel topography of human alpha EEG fields. Electroencephalogr Clin Neurophysiol.

[66] Duffy FH, Burchfiel JL, Lombroso CT (1979). Brain electrical activity mapping (BEAM): a method for extending
the clinical utility of EEG and evoked potential data. Ann Neurol.

[67] Jelic V, Kowalski J (2009). Evidence-based evaluation of diagnosticaccuracy of resting EEG in
dementia and mild cognitive impairment. Clin EEG Neurosci.

[68] Liedorp M, van der Flier WM, Hoogervorst EL, Scheltens P, Stam CJ (2009). Associations between patterns of EEG abnormalities and diagnosis
in a large memory clinic cohort. Dement Geriatr Cogn Disord.

[69] Loeches MM, Gil P, Jimenez F (1991). Topographic maps of brain electrical activity in primary
degenerative dementia of Alzheimer type and multi-infarct
dementia. Biol Psychiatry.

[70] Saletu B, Paulus E, Grunbergerer J, Maurer K (1993). Correlation maps: on the relation of electroencephalographic slow
wave activity to computerized tomography and psycopathometric measurements
in dementia. Imaging of brain in psychiatry and related fieldsed.

[71] Pucci E, Belardinelli N, Cacchiò G, Signorino M, Angeleri F (1999). EEG power spectrum differences in early and late onset forms of
Alzheimer's disease. Clin Neurophysiol.

[72] Dierks T, Perisic I, Frölich L, Ihl R, Maurer K (1991). Topography of the qEEG in dementia of Alzheimer type: relation to
severity of dementia. Psychiatry Res.

[73] Leuchter AF, Cook IA, Newton TF (1993). Regional differences in brain electrical activity in dementia:
use of spectral power and spectral ratio measures. Electroencephalogr Clin Neurophysiol.

[74] Anderer P, Saletu B, Klöppel B, Semlitsch HV, Werner H (1994). Discrimination between demented patients and normals based on
topographic EEG slow wave activity: comparison between z statistics,
discriminant analysis and artificial neural network
classifiers. Electroencephalogr Clin Neurophysiol.

[75] Nielsen T, Montplaisir J, Lassonde M (1993). Decreased interhemispheric EEG coherence during sleep in agenesis
of the corpus callosum. Eur Neurol.

[76] Leuchter AF, Spar JE, Walter DO, Weiner H (1987). Electroencephalographic spectra and coherence in the diagnosis of
Alzheimer's-type and multi-infarct dementia. Arch Gen Psychiatry.

[77] Fonseca LC (2008). Demência: eletroencefalograma e eletroencefalograma
quantitativo.

[78] Miyauchi T, Hagimoto H, Ishii M (1994). Quantitative EEG in patients with presenile and senile dementia
of the Alzheimer type. Acta Neurol Scand.

[79] Dierks T, Frolich L, Ihl R, Maurer K (1995). Correlation between cognitive brain function and electrical brain
activity in dementia of Alzheimer type. J Neural Transm Gen Sect.

[80] Goate A, Chartier-Harlin MC, Mullan M (1991). Segregation of a missense mutation in the amyloid precursor
protein gene with familial Alzheimer's disease. Nature.

[81] Sherrington R, Rogaev EI, Liang Y (1995). Cloning of a gene bearing missense mutations in early-onset
familial Alzheimer's disease. Nature.

[82] Levy-Lahad E, Wasco W, Poorkaj P (1995). Candidate gene for the chromosome 1 familial Alzheimer's disease
locus. Science.

[83] Wattamwar PR, Mathuranath PS (2010). An overview of biomarkers in Alzheimer's disease. Ann Indian Acad Neurol.

[84] Bertram L, Tanzi RE (2005). The genetic epidemiology of neurodegenerative
disease. J Clin Invest.

[85] Saunders AM, Strittmatter WJ, Shemechel D (1993). Association of apolipoprotein E allele epsilon 4 with late-onset
familial and sporadic Alzheimer's disease. Neurology.

[86] Strittmatter WJ, Saunders AM, Shemechel D (1993). Apolipoprotein E: high-avidity binding to beta-amyloid and
increased frequency of type 4 allele in late-onset familial Alzheimer
disease. Proc Natl Acad Sci U S A.

[87] Patterson C, Feightner JW, Garcia A, Hsiung GY, MacKnight C, Sadovnick AD (2008). Diagnosis and treatment of dementia: 1. Risk assessment and
primary prevention of Alzheimer disease. CMAJ.

[88] Andrade FM, Larrandaburu M, Callegari-Jacques SM, Gastaldo G, Hutz MH (2000). Association of apolipoprotein E polymorphism with plasma lipids
and Alzheimer's disease in a Southern Brazilian population. Braz J Med Biol Res.

[89] Schwanke CH, da Cruz IB, Leal NF, Scheibe R, Moriguchi Y, Moriguchi EH (2002). Analysis of association between APOE polymorphism and
cardiovascular risk factors in an elderly population with
longevity. Arq Bras Cardiol.

[90] Fernandez LL, Scheibe RM (2005). Is MTHFR polymorphism a risk factor for Alzheimer disease like
APOE?. Arq Neuropsiquiatr.

[91] Souza DR, de Godoy MR, Hotta J (2003). Association of apolipoprotein E polymorphism in late-onset
Alzheimer's disease and vascular dementia in Brazilians. Braz J Med Biol Res.

[92] Bahia VS, Kok F, Marie SN, Shinjo SO, Caramelli P, Nitrini R (2008). Polymorphisms of APOE and LRP genes in Brazilian individuals with
Alzheimer disease. Alzheimer Dis Assoc Disord.

[93] Bird TD (2008). Genetic aspects of Alzheimer disease. Genet Med.

[94] Bertram L, McQueen MB, Mullin K, Blacker D, Tanzi RE (2007). Systematic meta-analyses of Alzheimer disease genetic association
studies: the AlzGene database. Nat Genet.

[95] Rogaeva E, Ming Y, Lee JH (2007). The neuronal sortilin-related receptor SORL1 is genetically
associated with Alzheimer disease. Nat Genet.

[96] Lee JH, Cheng R, Schupf N (2007). The association between genetic variants in SORL1 and Alzheimer
disease in an urban multiethnic community-based cohort. Arch Neurol.

[97] Butler AW, Ng NY, Hamshere ML (2009). Meta-analysis of linkage studies for Alzheimer1s disease-a web
resource. Neurobiol Aging.

[98] Wollmer MA (2010). Cholesterol-related genes in Alzheimer's disease. Biochim Biophys Acta.

[99] Eschweiler GW, Leyhe T, Klöppel S, Hüll M (2010). New developments in the diagnosis of dementia. Dtsch Arztebl Int.

[100] Ashida S, Koehly LM, Roberts JS (2009). Disclosing the disclosure: factors associated with communicating
the results of genetic susceptibility testing for Alzheimer
disease. J Health Commun.

[101] Bekris LM, Yu CE, Bird TD, Tsuang DW (2010). Genetic of Alzheimer disease. J Geriatr Psychiatry Neurol.

[102] Taner NE (2007). Genetics of Alzheimer disease: a centennial
review. Neurol Clin.

[103] Ashida S, Koehly LM, Roberts JS (2010). The role of disease preceptors and results sharing in
psychological adaptation after genetic susceptibility testing: the REVEAL
Study. Eur J Hum Genet.

[104] Williamson J, Goldman J, Marder KS (2009). Genetic aspects of Alzheimer disease. Neurologist.

[105] Chung WW, Chen CA, Cupples LA (2009). A new scale measuring psychological impact of genetic
susceptibility testing for Alzheimer disease. Alzheimer Dis Assoc Disord.

